# Compact and Scalable Large Vortex Array Generation Using Azocarbazole Polymer and Digital Hologram Printing Technique

**DOI:** 10.1186/s11671-022-03675-7

**Published:** 2022-04-05

**Authors:** Boaz Jessie Jackin, Masaki Shirai, Honoka Haginaka, Kenji Kinashi, Naoto Tsutsumi, Wataru Sakai

**Affiliations:** 1 Materials Innovation Laboratory, Kyoto Institute of Technology, Kyoto, Japan; 2 Master’s Programs of Materials Chemistry, Kyoto Institute of Technology, Kyoto, Japan; 3 Bachelor’s Program of Materials Chemistry, Kyoto Institute of Technology, Kyoto, Japan; 4 Faculty of Materials Science and Engineering, Kyoto Institute of Technology, Kyoto, Japan

**Keywords:** Optical vortex, Computer-generated holography, Hologram printing, Photoinduced birefringence, Optical integrated device

## Abstract

An integrated device capable of generating large number of multiplexed optical vortex beams with arbitrary topological charge is considered as one of the crucial requirement for driving information photonics forward. Here we report a simple method for simultaneous generation of 100 multiplexed optical vortex beams from a polymer film of size 1 mm^2^ and thickness of 30 μm. This is achieved through a combination of computer-generated holography, digital hologram printing and photoisomeric polymers. When the fabricated sample is illuminated with a collimated laser beam, a pre-determined vortex array with arbitrary topological charge is emitted. The polymer film easy to synthesize and exhibits a diffraction efficiency of 30% with a retention period longer than 50 days.

## Introduction

Though photonics offers higher speed, large parallelization, low noise and low energy consumption compared to their electronic counterparts, they have enjoyed limited success in information processing applications. The main reason is the bulky system resulting from the assembly of free-space optical components (lenses, mirrors, beam-splitters, light modulators, etc.) on an optical bench. This leads to the cost and the size footprint of the system to increase nonlinearly with system performance (in our case the space-bandwidth product) and hence lacks scalability. Optical vortex beam generators (especially vortex array generators) are no exception to the above snag. Hence photonics technology demands a transition from the existing table-top format systems to an on-board or on-chip format system that is capable of driving the system footprint to scale linearly with the performance output. In other words, a two-time increase in system performance (space-bandwidth product) incurs a multiple time (nonlinear) increase in system size and cost for a free-space optical system, whereas an integrated system just incurs a two-time (linear) increase in size and cost. This requires novel materials and methods for denser integration of optical functions and has resulted in the emergence of a new field on its own known as ‘Integrated optics’. Optical vortex generators have also undergone significant improvements in optical integration in the past decade. In this paper, we report a simple and novel technique for generation of optical vortex array from a hologram of size 1 mm^2^. Compared to existing methods (discussed below), the reported method is capable of generating a large array of 100 vortex beams with a significantly small size footprint.

### Integrated Vortex Array Generators

The class of optical vortex generators that emit multiple optical vortex beams simultaneously from a single input beam has attracted huge interest in the information processing community. These multiple optical vortice emissions take any one of the following form: i) each emitted vortex beam takes a unique pre-defined position in space and is spatially separated from each other known as a vortex array or vortex lattice, and ii) all vortex beams exist co-axially and are decoupled at the detection stage using a mode-sorting or mode de-multiplexing arrangement. ‘Vortex arrays’ are more beneficial for information processing operations since each vortex beam in the array can independently interact in space with a medium/other vortex and be modulated individually to realize a required function. On the other hand, co-axially emitted modes are useful for information transmission as they can be coupled into fiber or launched into free space. Computer-generated holography (CGH) using spatial light modulators (SLM) are the most popular technique used for simultaneous generation of large number of multiplexed optical vortex beams [1, 2]. These systems are assembled on an optical bench making it significantly bulky and non-scalable. Several techniques for miniaturization and optical integration were developed and reported in the past decade. In spite of the significant efforts, generation of vortex array with array sizes and topological charges sufficient to realize computation operations has not been realized yet. The following presents details on what has been achieved so far and the existing limitations.

### Silicon-on-Insulator (SOI)

Considering multiplexed optical vortex generators, most of the research on silicon photonics are focused on emission of co-axially superposed vortex beams [3, 4], which are suitable for communication purposes. There are only a few existing reports that demonstrate optical vortex array generation. Cai et al. [5] have reported multiple vortex beam generation by fabricating silicon micro-ring resonators which support whispering gallery modes (WGM) carrying orbital angular momentum (OAM). The input light is coupled into the micro-rings through a waveguide, and the generated vortex beams were coupled out to free space by inscribing angular grating structures on the inner wall of the ring structures. The device is OAM tunable by changing the injected wavelength and operates in the range of 1470 nm to 1580 nm. A 1D array of 3 vortex beams were generated by fabricating three identical ring structures on the same substrate. Optical vortex lattice generation using the principle of ‘three-plane wave interference’, implemented by fabricating three parallel waveguides with etched tilt gratings on a SOI platform has been reported by Du et al. [6]. Though silicon photonics has the advantage of advanced tooling (from the electronic industry), large optical vortex array generation (100 or larger OAM modes) has not been reported yet. Moreover, silicon photonics works only in telecommunication wavelength (1.5 μm to 2.5 μm), whereas a lot of interesting optical OAM events/phenomena occur in the visible wavelength (400 nm to 700 nm) regime.

### Metasurface

Metasurfaces, also known as 2D metamaterials, are a distribution of sub-wavelength structures, either inscribed or deposited on a 2D substrate. A plasmonic metasurface fabricated using an array of gold nanoantennas and capable of simultaneously emitting four optical vortex beams with different topological charges ranging from *l*=1 to *l*=4 was reported by Yue et al. [7]. Maguid et al. have combined the properties of harmonic response and geometric phase of metamaterials to fabricate a harmonic response geometric phase metasurface [8]. Six vortex beam modes with topological charges *l*=1,2,3,-1,-2,-3 (harmonics) are generated when the metasurface is illuminated with a polarized Gaussian beam. Metasurface fabricated by milling nanoholes in gold substrate capable of modulating both the amplitude and phase of the input beam was reported by [9]. A 6 × 6 array of vortex beam with topological charge ranging from *l* = − 4 to *l*=3 was demonstrated. Ren et al. have introduced the concept of OAM conserving meta-hologram where the output is a OAM-pixelated image [10]. The metasurface when illuminated with a OAM beam reconstructs a OAM-pixelated image where each pixel possesses a topological charge in accordance with the charge of input beam. Multiplexing multiple vortex arrays were recently reported by Jin et al. [11]. Multiple optical vortex arrays were angularly multiplexed using the principle of superposition of holograms and are implemented using a geometric metasurface. Three distinct vortex arrays (*l* = − 2 to *l* = 3) were generated from the same metasurface when illuminated from three different angles. The vortex arrays discussed so far had a 2D profile (each vortex beam in the array is arbitrarily positioned in the lateral (x-y) plane), but Huang et al. reported the possibility of generating a 3D vortex array (where the profile of each vortex beam can be made to arbitrarily vary in the longitudinal (z) axis) [12]. They fabricated a dielectric metasurface which was able to generate a 5 × 5 3D vortex array (*l* = − 8 to *l* = 0) which exhibits different beam profile in the z-direction. Another 3D optical vortex array generation method that is sensitive to the input wavelength was reported by Jin et al. [13]. Two different 3 × 3 3D vortex arrays were generated from the same metasurface only by switching wavelengths. Reflection type metasurfaces, known as ‘meta-reflectarray’, was used for vortex array generation by Liu et al. [14], where 5x5 vortex array with topological charge varying from *l* = − 4 to *l* = + 12 was successfully generated. The reflection configuration helps to achieve a maximum diffraction efficiency of $~70\%$ and broad bandwidth sensitivity ranging from 1250 to 1750 nm. To the best of our knowledge, generation of a large vortex array ($\sim$100) with higher topological charges has not been reported yet. The reason can be attributed to the challenges in fabricating sub-wavelength structures that could execute larger phase modulation, higher harmonic generation or extended spin-orbit angular momentum exchange.

### Computer-Generated Holography

Here we discuss the existing techniques for optical vortex array generation based on computer-generated holography (CGH) that are scalable and posses a small size footprint and are eligible for optical integration. Zhuang et al. [15] have reported the generation of vortex array of size 3 × 3 with topological charge ranging from *l* = − 2 to *l* = 3 using a micro-CGH fabricated on lithium niobate crystal using direct laser writing. Emission of optical vortex array from holographically recorded vortex lattices on nematic liquid crystal cells [16] and polymer-dispersed liquid crystal films [17] has also been reported. Here an array of circularly polarized beams is used to generate a corresponding array of vortex beams from the hologram. It is also worth discussing few single vortex generators based on CGH, as they are very close to the proposed work. Carpentier et al. [18] have reported generation of optical vortex of charge *l*=1 to *l*=4 using a CGH of size 5 mm^2^ recorded on a photographic film using optical reduction. Computer-generated holograms recorded inside glass substrate by femtosecond laser pulses for generation of optical vortices were reported by Guo et al. [19]. Large vortex array generation employing scalable and compact CGH’s has not been reported yet.

The discussions from above paragraphs can be summarized as follows: i) the reported SOI-based methods require light coupling into individual micro-resonators which results in significant challenges in fabricating a device for large optical vortex array generation, ii) metamaterials are the most reported devices for integrated vortex array generation but they suffer from limited phase modulation capabilities, resulting in vortex beams of small topological charges and a small array size. iii) CGH’s are often reported as a bulk device employing an SLM for optical vortex array generation. Some reports present CGH’s as an integrated device but are limited in vortex array size and topological charge. It can be concluded that an integrated devices capable of generating a large optical vortex array with large topological charges has not been reported yet. But as mentioned earlier, such an integrated device is one of the key requirement in practically realizing an optical information processing machine. In this paper, we report a very simple yet efficient CGH-based method that can emit a $10 \times 10$ vortex array of topological charge *l*=10 from polymer sample size of 1 mm^2^.

## Material and Methods

**Fig. 1:**
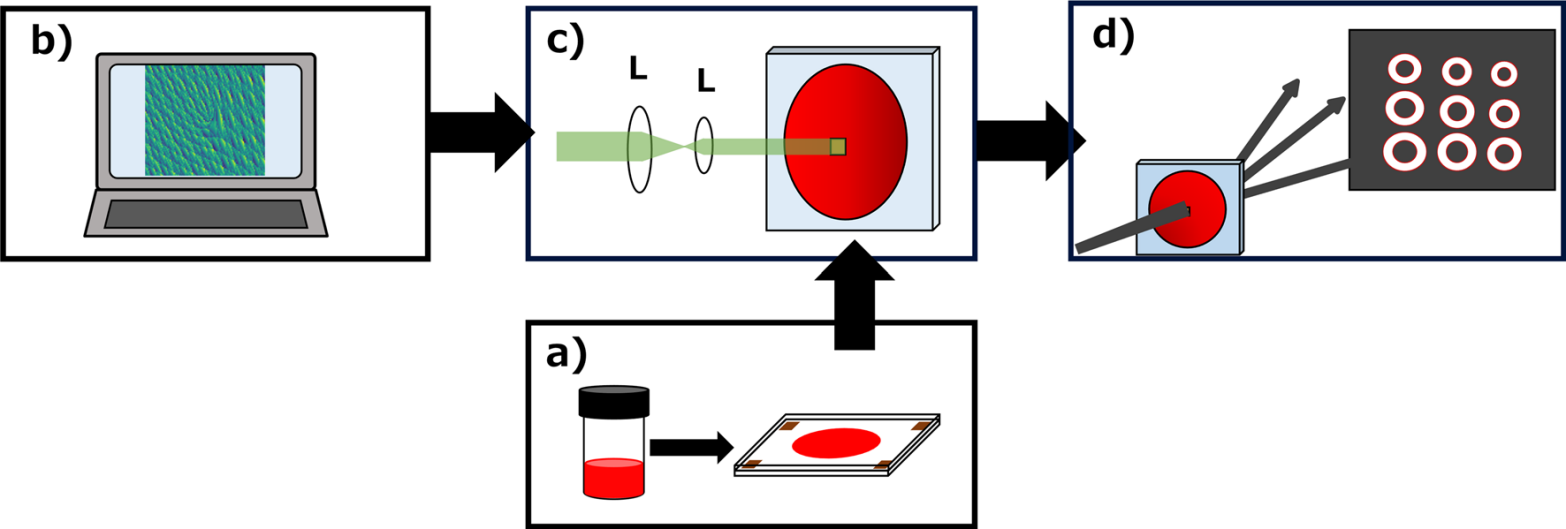
Schematic showing the steps involved in devising the optical vortex array generator, **a** synthesis of hologram material (Azocarbazole polymer), **b** digital computation of hologram pattern capable of reconstructing the vortex array, **c** optical transfer of the computed pattern onto the synthesized material (digital hologram recording) and **d** reconstruction of optical vortex array by illuminating the recorded digital hologram using a Gaussian beam

The purpose here is to record computer-generated hologram on a small area of a polymer film, which (later) when illuminated with a Gaussian beam emits an array of optical vortex beams with arbitrary topological charges. The above-mentioned is executed as a four-step process consisting of (a) sample synthesis, (b) hologram computation, (c) hologram printing and (d) hologram reconstruction, as shown in Fig. 1. The material chosen for hologram recording is Azocarbazole polymer which is fabricated as a thin film. The computed hologram pattern is a superposition of multiple fork gratings each of which correspond to a single optical vortex beam. The computed pattern is then optically transferred to the polymer film as refractive index modulation. The sample when illuminated with a Gaussian beam emits an array of vortices in accordance with the recorded pattern. Each step in the process is explained below,

### Photoisomerization

**Fig. 2:**
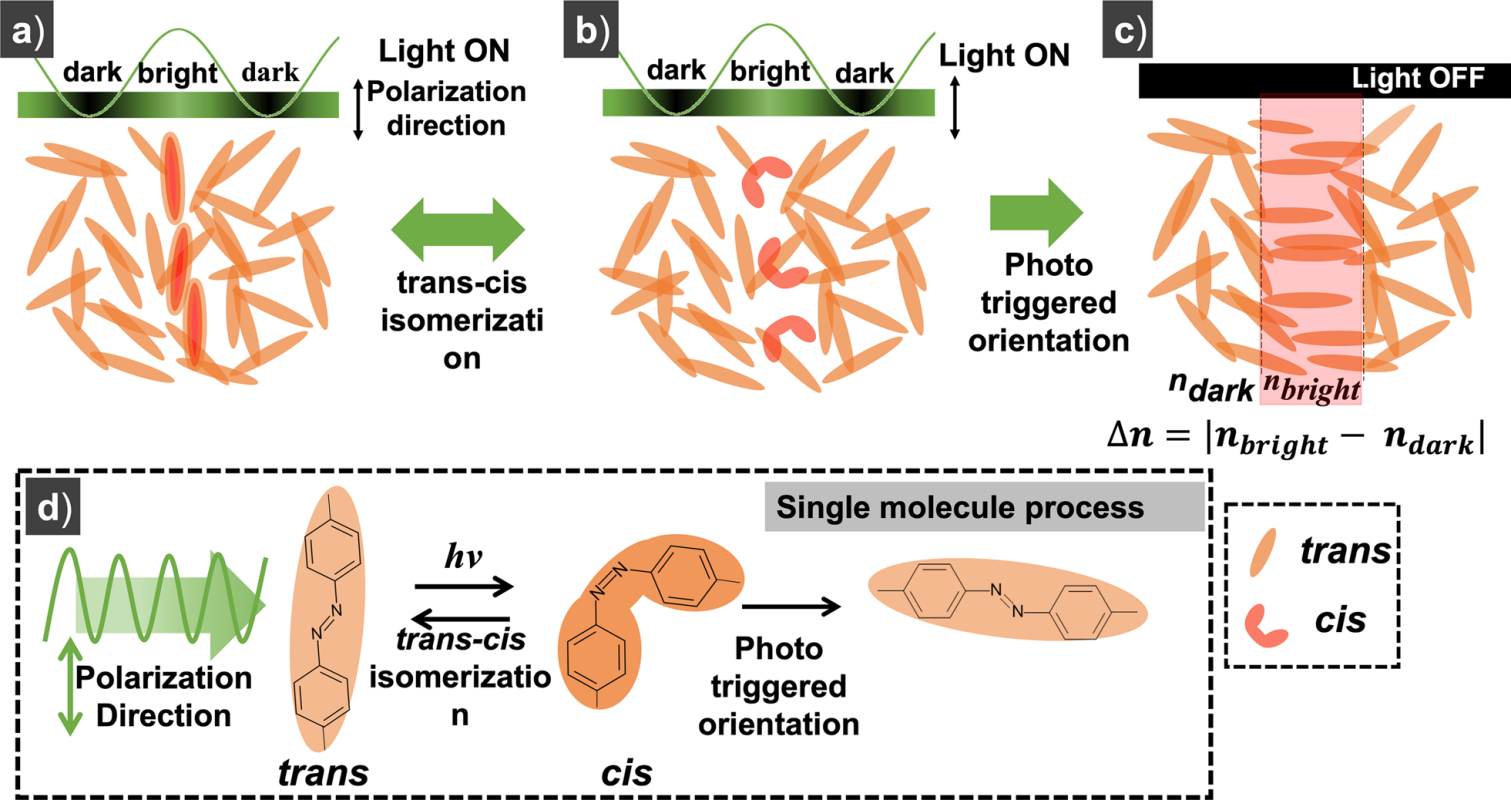
Schematic showing the process involved in the transfer of light intensity pattern as refractive index modulations ($\Delta n$) on the material sample utilizing the phenomena of photoinduced isomerization. **a**, **b** Cis-trans isomerization cycle, **c** phototriggered orientation. **d** Schematic of the photoinduced isomerization process at a single molecule level

The sample being prepared to be used for hologram recording works on the principle of photoinduced birefringence. This sample material when exposed to light radiation exhibits photoisomerization by switching between a trans and cis state known as trans-cis isomerization. When illuminated with a light pattern with alternate bright and dark regions (fringe pattern), only the molecules in the sample that are exposed to light radiation (bright region) undergo isomerization as shown in Fig. 2a, b. Once the light is turned off, these exposed molecules get oriented in a direction perpendicular to the polarization of the input light beam as shown in Fig. 2c. These orientations remain intact until perturbed again by heat or a strong radiation, and hence, the recorded change is permanent. The recorded molecular orientation induces anisotropy, and hence, the material becomes birefringent in the exposed region. So a light polarized in a suitable direction experiences a difference in refractive index when passing through the exposed (oriented) and un-exposed areas. Thus by spatially modulating the incident light intensity distribution in accordance with the pattern to be transferred, and by exposing the sample onto the radiation, the corresponding pattern is recorded on the material as refractive index modulation ($\Delta n$) as shown in Fig. 2c.

### Sample Synthesis

**Fig. 3:**
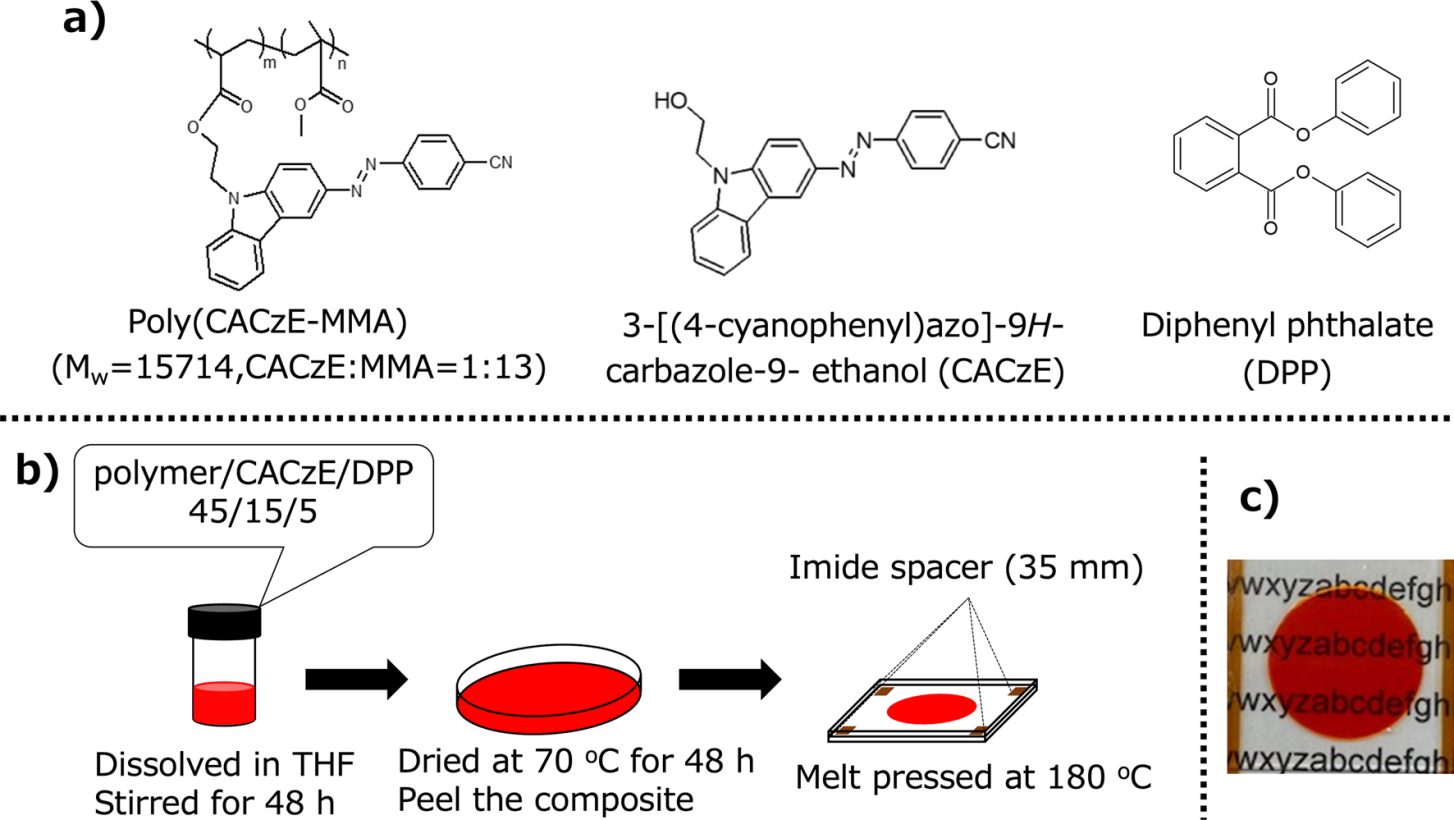
**a** Chemical structure of the materials used for sample preparation, **b** (left to right) the three steps involved in the polymer film fabrication and **c** photograph of the fabricated sample placed on top of printed alphabets, showing good transmittance

The material that serves as the photoisomeric medium in this experiment is an azoderivative called as azocarbazole. It is made of an azobenzene-functionalized copolymer, poly(CACzE-MMA), which is composed of 3-[(4-cyanophenyl)azo]-9H-carbazole-9-ethanol (CACzE), methyl methacrylate (MMA), CACzE and diphenyl phthalate (DPP), as shown in Fig. 3a. The procedure for synthesis of poly(CACzE-MMA) and CACzE was reported earlier by Kinashi et al. [20]. DPP was purchased from a commercial source (Tokyo Kasei Co.) and used as a plasticizer to decrease the glass transition temperature $T_g$ of the azocarbazole composite. In this study, the composition of polymer/CACzE/DPP was fixed at 45/15/5 by weight. The mixture was first stirred in tetrahydrofuran (THF) for 48 h and then cast on a hot plate at $70^o$C for another 48 h. The resulting polymeric powder was pressed between two glass substrates at $180^o$C as shown in Fig. 3b. Polyimide (or Teflon) spacers were used to adjust the thickness of the working layer to be 35 μm. The size of the sample was 3 cm × 3 cm, and a photograph of the synthesized sample is shown in Fig. 3c. The procedure mentioned by Kinashi et al. [20] was used to experimentally measure the haze value of the sample. Haze value is a measure of the transparency of the sample and was found to be 1.2 at 633 nm wavelength, which indicates high transparency. The stability of the recorded grating pattern (retention time) for the synthesized sample was experimentally obtained using the procedure reported earlier [20]. The measured retention rate was $70\%$ after a period of 50 days, which indicates good stability at room temperatures.

### Hologram Computation

**Fig. 4:**
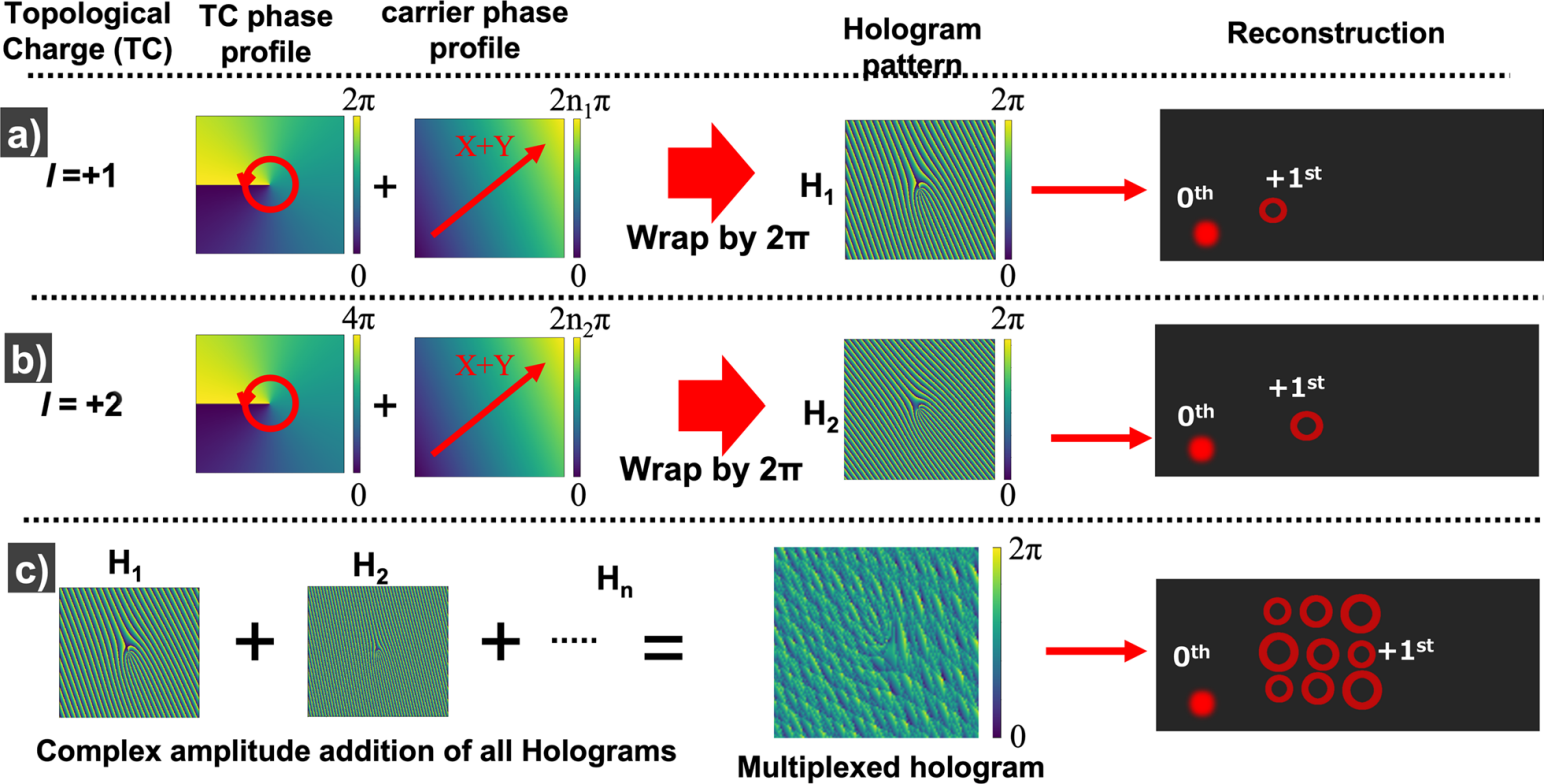
**a** (left to right) The spiral phase profile corresponding to TC $l= +1$ is added to a linear phase profile (carrier) and then wrapped by $2\pi$ to generate the hologram pattern $H_1$, which then reconstructs the optical vortex beam of TC $l=+1$, **b** the spiral phase corresponding to TC $l=+2$ is added to a linear phase profile (carrier) and then wrapped by $2\pi$ to compute the hologram pattern $H_2$, which then reconstructs the optical vortex beam of TC $l=+2$, **c** The phases of holograms $H_1, H_2, \ldots , H_n$ are added together to generate the multiplexed hologram, which when reconstructed emits all the corresponding vortices as an array

Since the amplitude and phase of a vortex beam can be well-defined as a mathematical function, numerical simulation or diffraction calculation (as in the case of conventional holography) is not required. Instead, it is simply required to construct 2D matrices that represent the phase of the vortex beam cross section (spiral phase) and a carrier beam cross section (linear phase). Adding these two matrices and wrapping them by $2\pi$ gives rise to the hologram pattern $H_1$ (also known as fork grating) as shown in Fig. 4a, b. This hologram is known as the ‘phase hologram’ and is a simple hologram encoding technique commonly used. More complex hologram encoding techniques that offer higher mode purity has also been reported [21]. The computed phase hologram can also be encoded as a Damman grating which reduces the hologram to a binary pattern and also enables equal distribution of power into multiple diffraction orders. Considering the fact that i) the recording material being used here is capable of recording greyscale patterns and ii) only the first-order diffracted beam is of interest, we adapt the simple phase hologram encoding technique in this work. The phase gradient of the carrier beam defines the position where the vortex beam will be reconstructed. (Vortex beams are observed as a donut-shaped intensity distribution and hence are represented as circular rings in Fig. 4). The steeper the phase gradient of carrier beam larger will be the diffraction angle during reconstruction. This results in the vortex beam being reconstructed farther from the 0th-order beam. This is graphically presented in Fig. 4, where sub-figure (b) represents a carrier with larger phase gradient than the one in sub-figure (a). Hence the reconstructed optical vortex shown in sub-figure(b) is located at a farther position from the 0th order, compared to the optical vortex in sub-figure(a). Thus by choosing an appropriate carrier beam to be added with each vortex beam, an array of non-overlapping vortex beams can be created. In other words, each vortex beam emerges from the hologram at a slightly different angle during reconstruction and is also known as ‘angular multiplexing’. Multiple holograms ($H_1,H_2,\ldots H_n$) can be computed by adding each vortex beam with a different carrier beam. Adding all these multiple holograms together ($H_1 + H_2 + \cdots + H_n$) gives rise to a single multiplexed hologram as shown in Fig. 4c. The multiplexed hologram is capable of reconstructing each of the recorded vortices in its appropriate position as a 2D array (Fig. 4c).

### Digital Hologram Printing

**Fig. 5:**
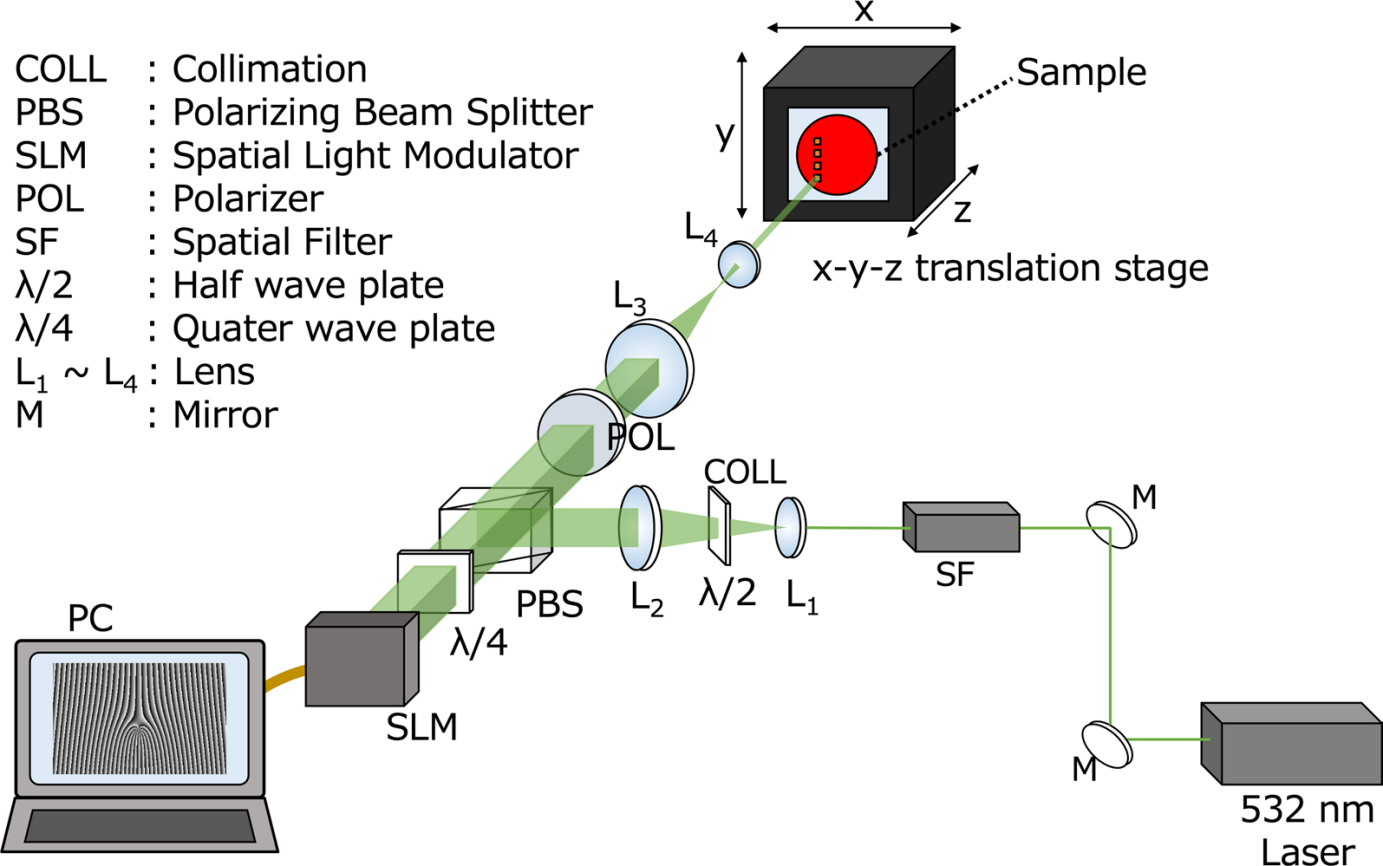
Schematic of the optical printing set-up built on the optical table for digital hologram printing

The optical set-up used to transfer the computed hologram onto the fabricated polymer film is shown in Fig. 5. The beam from a laser source of wavelength 532 nm is filtered and collimated and send into an SLM. The SLM displays the computed hologram pattern and the beam reflected back from the SLM is modulated accordingly in amplitude. The SLM used is Holoeye LC-R 1080 which is configured to work in amplitude only mode using a combination of quarter wave plate and polarizing beam splitter (PBS) as shown in Fig. 5. The sample is mounted on a three-axis translation stage at a distance parallel to and facing the SLM. The light reflected from the SLM reaches sample through a set of de-magnifying and imaging lenses. Though only two lenses ($L_3 \& L_4$) are shown in Fig. 5 for simplicity, it should be noted that is actually composed of 7 lens elements to cancel out aberrations. It is this imaging optics that enables the efficient transfer of the pattern displayed by the SLM onto the sample with a 10x de-magnification. Raytracing simulations (zemax software) showed the spot size on the imaging plane to be 0.8 μm with a tolerance of 20 μm in focal shift. So it is necessary to place the sample at the focal plane of the lens with an accuracy of 20 μm. This is realized by mounting the sample on a high accuracy motorized 3-axis motion stage equipped with a encoder-based feedback. The power of the laser beam at the recording plane was 32 mW/mm^2^ and the exposure time for recording is 5 s (single exposure). The recorded refractive index modulations on the sample were then verified using a phase contrast microscope.

**Fig. 6:**
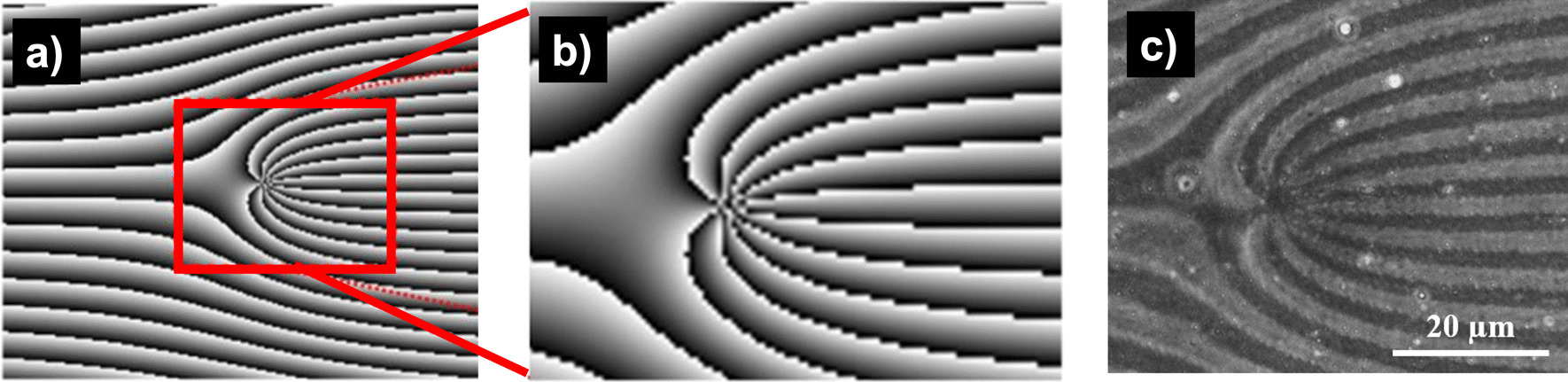
**a** The digitally computed hologram pattern corresponding to topological charge $l=-10$, **b** magnified image of the region of interest from the center, **c** phase contrast microscopic image from the recorded sample corresponding to the region of interest

**Fig. 7:**
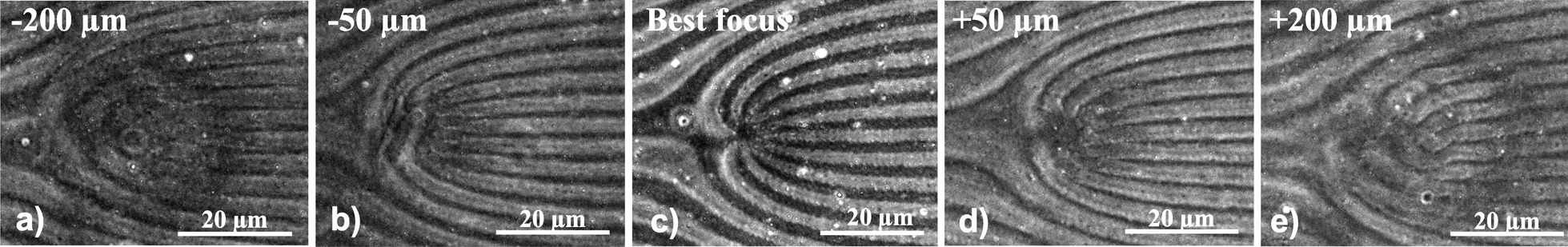
Phase contrast microscopic images (contrast enhanced) showing the printed accuracy being affected by sample position with respect to best focus. **a**, **d** 200 μm away from best focus, **b**, **d** 50 μm away from best focus and **c** at best focus

The computed fork grating of topological charge *l* = − 10 and the corresponding phase contrast microscopic images of sample after recording are shown in Fig. 6. Phase contrast microscopic images showing the effect of shift in sample position (from the best focus) on the accuracy of the printed hologram are shown in Fig. 7. Hence it is necessary to ensure accurate positioning of the sample with respect to the imaging optics, which is achieved by mounting the sample on a motion stage. The size of each pixel on the SLM is 8 μm which is de-magnified 10 times onto the polymer sample resulting in a pixel pitch of 0.8 μm. The displayed hologram consisted of 1200 × 1200 pixels which results in a printed hologram of size 0.960 mm × 0.960 mm in size. The printed pixel pitch and size were verified experimentally to be in agreement with the numbers mentioned above, through microscopic measurements.

## Results

**Fig. 8:**
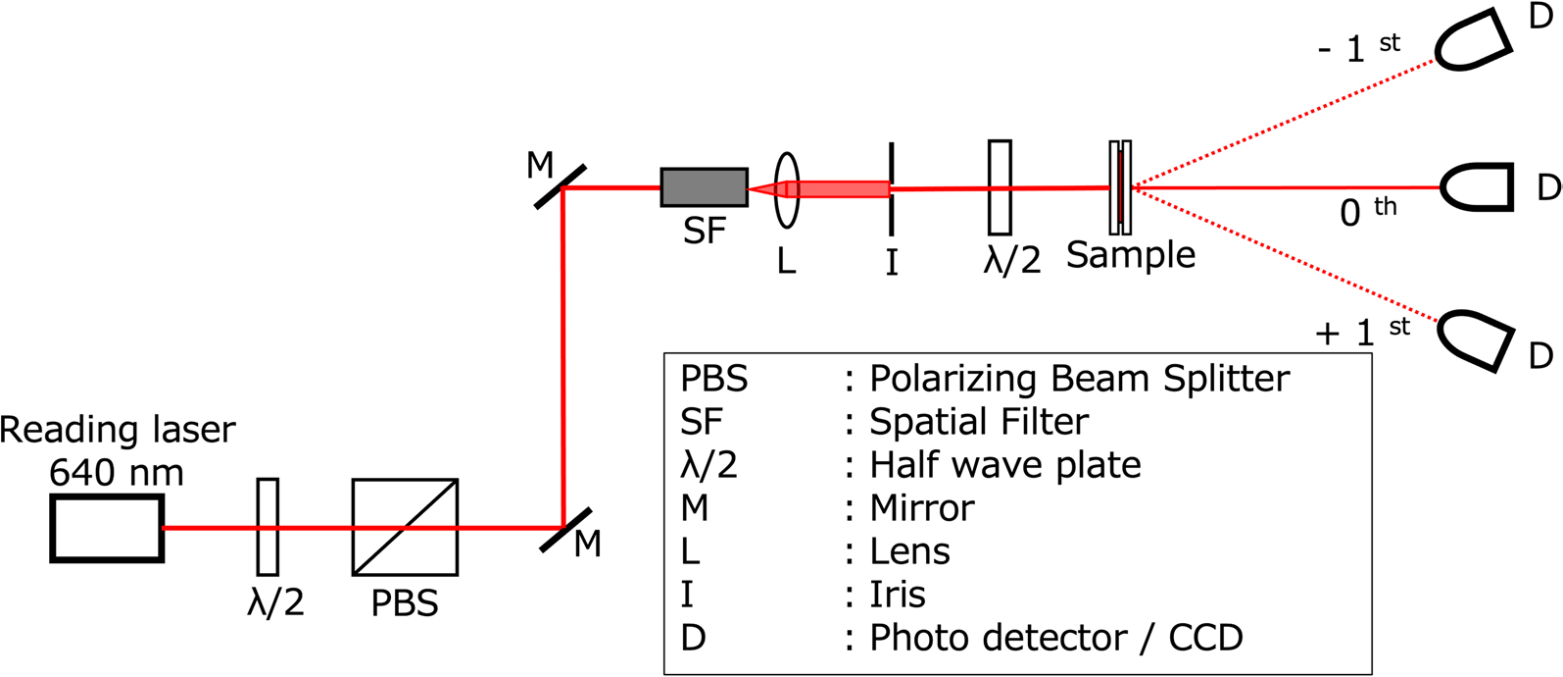
Schematic of the optical set-up used for reconstructing the printed holograms and for capturing the reconstructed light intensity pattern

**Fig. 9:**
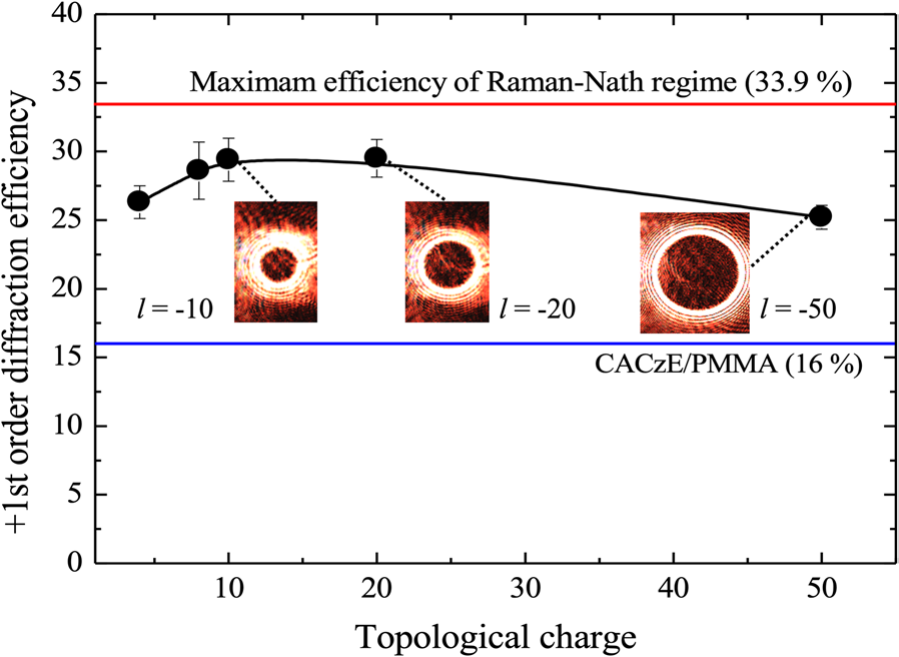
The plot of diffraction efficiency with different topological charge of the optical vortex beam. Diffraction efficiency close to the theoretical maximum for Raman-Nath regime (red line) has been achieved

The reconstruction experiments were carried out using the optical system shown in Fig. 8. It is a simple system designed with the purpose of generating a collimated beam of diameter 1 mm that matches with the size of the hologram printed. Since the fabricated sample is highly transmissive in the red region, a laser source of wavelength 640 nm was used. Optical vortex beams are emitted in all diffraction orders ($-\infty \ldots \infty$) when illuminated since the hologram (grating) here functions a thin grating that belongs to the Raman-Nath regime. It should be noted that even though the sample film had a thickness of 35 μm, it does not qualify as a volume Bragg grating since the recording is done using a single beam in a imaging configuration. It is only the first-order diffracted that is of importance since it possesses most of the power and all of the information. The zeroth-order ($I_0$) and first-order ($I_{-1},I_{+1}$) diffracted beam intensity is measured using photodiodes, and the diffraction efficiency $\eta$ is calculated using the formula shown in Eq.1. Figure 9 is a plot of the diffraction efficiency with respect to TC of emitted vortex beams. It can be concluded that the diffraction efficiency achieved is close to the maximum theoretical limit of Raman-Nath regime and does not change with topological charge.

1 $$\begin{aligned} \eta = \frac{I_{+1}}{I_{+1}+I_0+I_{-1}} \end{aligned}$$

**Fig. 10:**
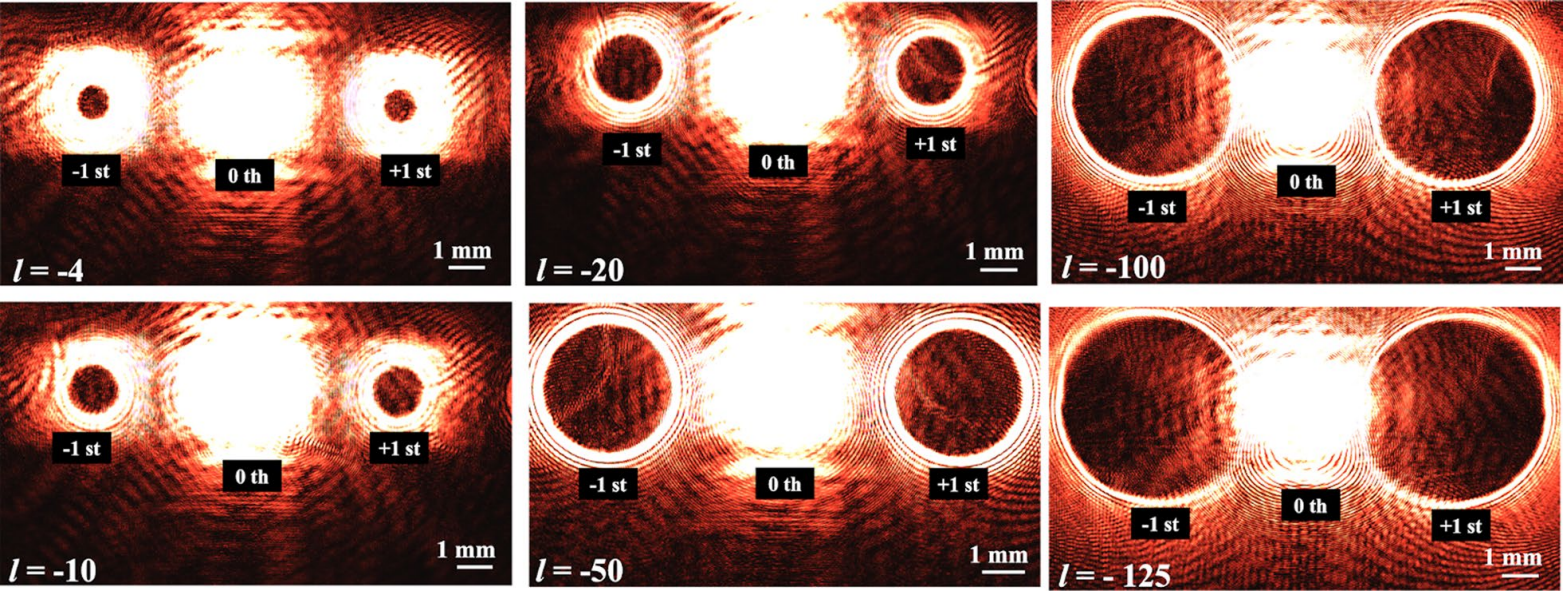
Reconstructions from single optical vortex beam generating holograms captured using a CCD sensor. The images show the 0th, +1st, -1st reconstructed diffraction orders for different topological charges (see embedded label for details)

**Fig. 11:**
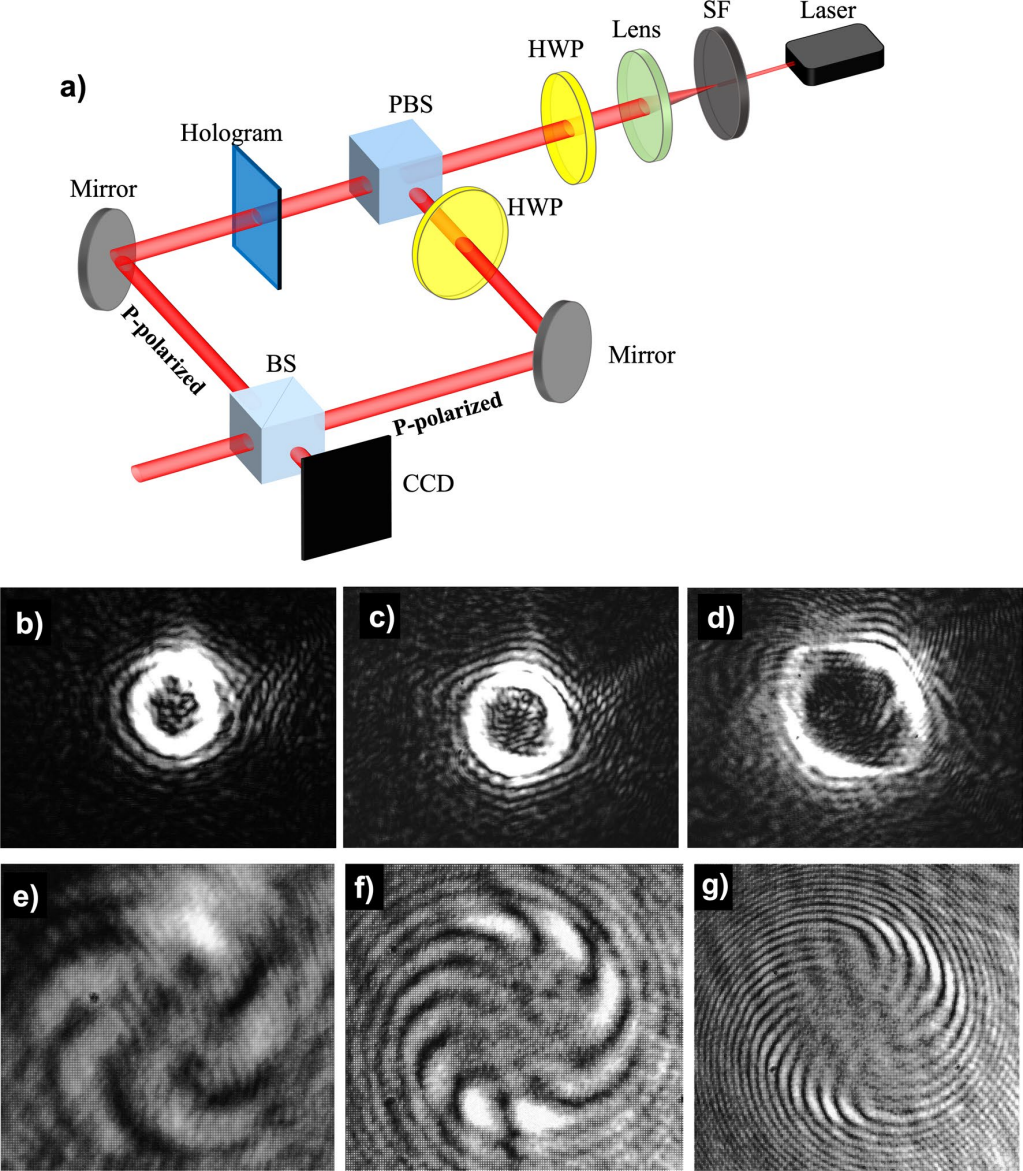
**a** Schematic of the optical set-up used to capture the interference pattern between Gaussian beam (with a small divergence) and optical vortex beam generated by a hologram. Intensity of vortex beams corresponding to topological charges **b** $l=-5$, **c** $l=-10$ and **d** $l=-25$ and their respective interference pattern **e** $l=-5$, **f** $l=-10$ and **g** $l=-25$ captured at the CCD plane. The number of fins in the interference pattern matches with the topological charge

Figure 10 shows the 0th and $1^{st}$ order diffracted beams captured using a charge-coupled device (CCD) sensor, from holographic reconstructions using the optical set-up shown in Fig. 8. The recorded pattern in these holograms correspond to a single vortex beam (not array) each with a different topological charge (*l* = − 4, − 10, − 20, − 50, − 100, − 125). The increase in radius of the reconstructed donut pattern with increasing topological charge can be observed from Fig. 10. It can also be concluded that the largest topological charge that can be reconstructed with high fidelity, from a single vortex (not array) hologram of size 1 mm^2^ is *l*=-50. In order to verify the topological charge of the reconstructed optical vortices an Mach–Zehnder-type interferometer was constructed as shown in Fig. 11. The interference pattern corresponding to the superposition of the generated vortex beam and a Gaussian beam (with a small divergence) was captured on the CCD sensor. The captured pattern that corresponds to vortex beam with topological charges *l* = − 5, − 10, − 25 is shown in Fig. 11b–d, respectively. The number of the fins in the interferograms matches with the topological charge of the vortex hologram used, which confirms the correctness of vortex beams generated.

**Fig. 12:**
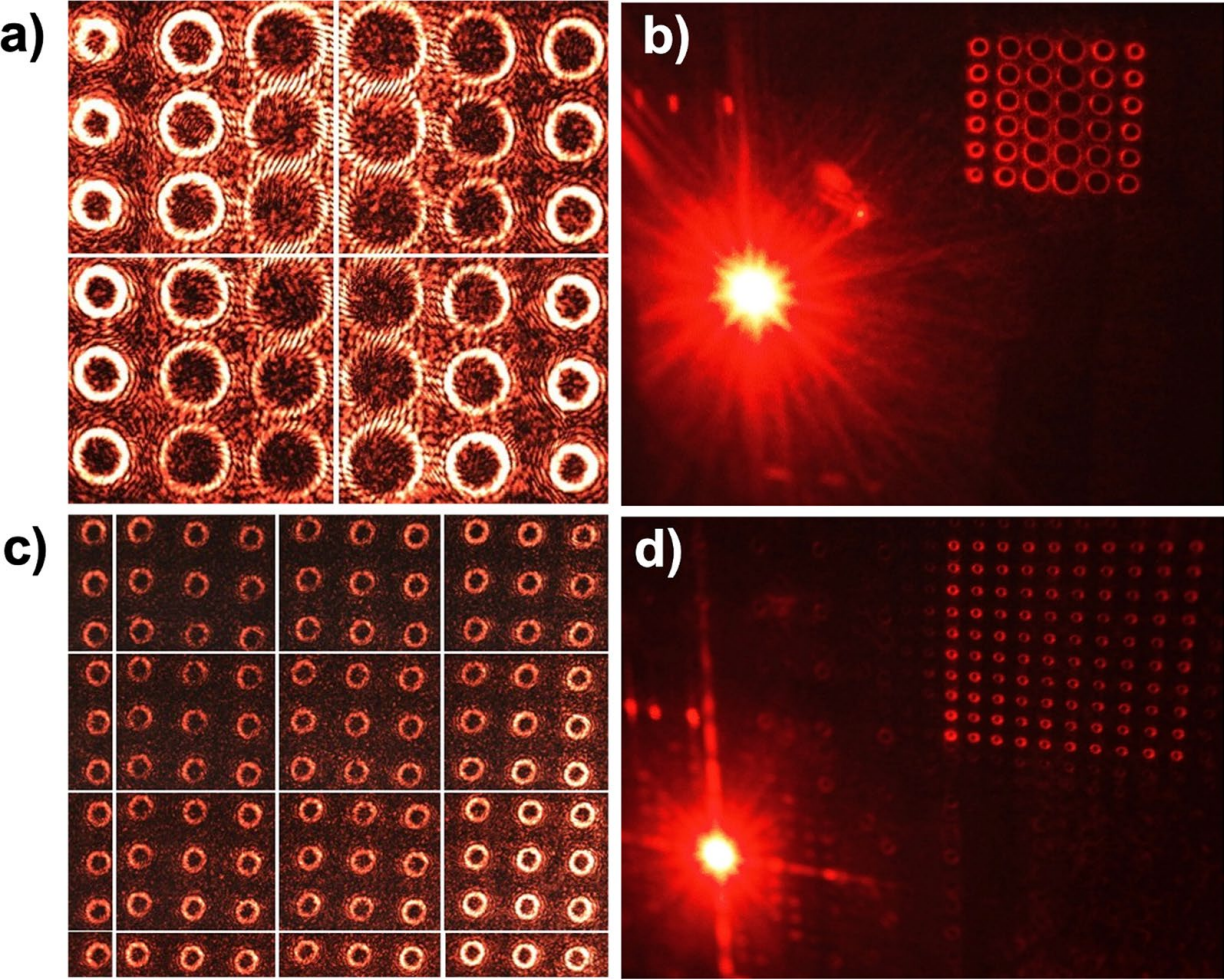
Reconstructions captured from +1st order diffracted beams of multiplexed holograms. A $6 \times 6$ optical vortex array consisting of topological charges $l=-6,-12,-24$ **a** captured directly on a CCD sensor, **b** captured from a screen using a camera showing the 0th order and +1st diffracted beams. A $10 \times 10$ optical vortex array consisting of topological charges $l=-10$ **c** captured directly on a CCD sensor, **d** captured from a screen using a camera, showing the 0th order and +1st diffracted beams.(The white horizontal and vertical lines in the CCD images (**a**, **c**) are due to a tiled capture procedure adapted due to the CCD sensor area being smaller than the reconstructed vortex array size)

Multiplexed hologram patterns corresponding to a $6 \times 6$ and $10 \times 10$ vortex array were recorded on the polymer and then subjected to reconstruction tests. Both the fabricated holograms had the same size 1 mm^2^ and the same number of pixels $1200 \times 1200$ mentioned earlier. The reconstructions were captured directly using a CCD camera at a distance of 5cm from the sample and are shown in Fig. 12a, c. The horizontal and vertical lines seen in the images are due to a tiled capture procedure adapted to circumvent the issue of vortex array size being larger than the CCD sensor area. Figure 12b, d represents the reconstructions captured from a black screen placed at a distance of 10cm from the hologram sample using a photographic camera. These images show the 0th-order and +1st-order reconstructed beams. A few donut-shaped pattern seen close to the 0th-order beam correspond to the weak autocorrelation term in holographic reconstruction. From the above experimental results, it can be concluded that a $10 \times 10$ optical vortex array with arbitrary topological charge up to *l* = − 10 can be successfully generated from a sample of size 1 mm^2^

## Discussion

### Maximum Array Size and Topological Charge

Hologram recording and reconstruction experiments were performed for larger array sizes than the one reported above ($10 \times 10$) upto $12 \times 12$. This resulted in an overlap of +1st and +2nd order reconstructed optical vortex arrays. So, it is required to use a carrier phase with a much steeper gradient in order to further separate out the +1st and +2nd diffraction orders, in order to avoid the overlap. This demands a smaller pixel pitch but the current hologram printing system is limited to a pixel pitch of 0.8 μm. The total number of vortex beam in the array also depends on the topological charge of each vortex. Since larger topological charges diverge fast and consumes larger space, it is required to reduce the number of vortices in the array in order to avoid overlap. The same applies to the largest topological charge that can be successfully generated. Beams with a large topological charge consume larger area and also exploit a steeper spiral phase gradient which in turn demands a smaller pixel pitch. Hence the topological charge is limited to $l=-50$ for a high-fidelity reconstruction in the reported system. The current limitations can be overcome to a certain extent by adapting a latest available SLM which boasts a pixel pitch of 3.75 μm.

### Further Possible Improvements

The currently reported diffraction efficiency can be further increased by adapting a volume hologram (thick hologram) recording configuration [22] . These are also called as Bragg holograms and requires the presence of another beam (as reference) during recording. Such a two beam recording can be performed using the currently reported optical system with minimal modification. Diffraction efficiency of $68\%$ has been reported for the same azocarbazole sample for such a two beam hologram recording at a wavelength of 532 nm [23]. Since only the $+1$ diffraction order is present while reconstructing a volume hologram, there will be no overlap (with +2nd and higher orders) which helps in increasing the number of vortices in array. It is also possible to further increase the number of pixels of the hologram further (from the currently reported $1200 \times 1200$), which could in turn increase the purity of optical vortex modes reconstructed. For this, a hologram stitching procedure is to be implemented by placing the sample on a x-y motion stage. Recording such larger stitched hologram to understand its effects on mode purity is one of the future goals in this research. The OAM of the generated beam must be quantified in order to match it with application requirements. The quantification studies reported so far used only a single vortex beam for measurement. But in an angularly multiplexed vortex array, each vortex beam emerges at a different angle from the hologram plane. Since the optical axis of each vortex beam points to a different direction, it is very challenging to realize a measurement system that provides results with high repeatability. Hence measurement of absolute OAM of each beam in the array and understanding its effect on array density demands extensive investigation. We intent to investigate the design and development of such a specially tailored system in the future. It is also important to note that the azo polymer used in this research exhibits photoinduced anisotropy through photoisomerization and molecular reorientation. The material anisotropy can induce geometric phase in the propagating beam and thereby opening up the possibility of vector beam mode generation. But a precise understanding and control of material anisotropy during recording is required.

### Dynamic Generation of Vortex Array

An important drawback of the proposed method is its inability to generate optical vortex arrays dynamically in real time as is done using an SLM. Though the reported azo polymer can be optically erased and rewritten, the re-writable speed is quite slow ($\tilde{5}$ s) for real-time operation [20]. Generally, information processing applications demand real-time updateable generation of beam modes which makes SLM as the most popular choice. But another strategy for dynamically generating modes is to do a static generation followed by a dynamic modulation. In other words, the generation process and dynamic modulation process should be separated out but be allowed to function together in order to realize dynamic generation. For example, optical functions such as multicasting, switching, coupling and mode mixing can be employed to generate any arbitrary vortex array pattern, from a basic set of fixed static vortex array generated. Integrated optics has shown significant progress in the above-mentioned direction and will be the key player in realizing such a strategy.

### Future Prospects of Optical Vortex Beams for Information Processing

As Moore’s law is closing-in on its saturation limit, several abstract computing paradigms (machine learning, artificial intelligence, etc.) are starting to feel the stress. These computing paradigms are currently implemented on the transistor-based von Neumann computing framework and an alternate framework is actively sought after. Photonics is considered as one of the promising candidate to succeed as an alternate framework. Today, photonics technology dominates electronics in the long-distance information transmission regime, but it struggles to catch up with electronics in the information processing regime. The main reason is the difficulty to ‘localize’ or confine photons (at the nanoscale) to a predetermined position in space and time, as we do with electrons (charge). So, photonics-based methods are generally used as an non-conventional information processing (computing) platform for tasks such as optical image processing, optical neural networks and information display, which do not demand localizing photons. Considering light, it is usually the amplitude, phase, wavelength and polarization degrees of freedom of light beams that are used as information carriers/modulators. Recent developments have revealed that the orbital angular momentum states (OAM) of light (known as vortex modes) is another degree of freedom and possesses better information processing potentials. The capabilities are attributed to its robust propagation characteristics and the unbound limit of orthogonal OAM states which helps in lossless transmission and the parallel processing of huge volumes digital data. The above-mentioned potentials, along with the availability of state-of-the-art fabrication techniques for optical vortex beam generators, have rekindled interest in the non-conventional optical computing frameworks mentioned earlier. We believe this research work will be a small stepping stone in this direction.

## Conclusion

A compact and scalable technique for optical vortex array generation is reported. The method utilizes digital hologram printing on azocarbazole polymer to record an optical vortex array of arbitrary shape and topological charge into hologram of size 1 mm^2^. This hologram when illuminated emits the recorded optical vortex array into the first-order diffracted beam. An array consisting of 10 × 10 optical vortices each with a topological charge of *l* = 10 has been achieved. The array size and topological charges can be further increased by stitching multiple holograms together using a tiled hologram printing technique. Here the size of stitched hologram increases linearly with the size of the array making the device ‘scalable’. Moreover, the size of the hologram is in the order of millimeter, making it ‘compact’ and suitable for optical integration. Hence the reported technique has the potential to serve as an integrated device in optical computing and communication applications.

## Data Availability

Not applicable.

## Abbreviations

CGH: Computer-generated holography
WGM: Whispering gallery modes
OAM: Orbital angular momentum
SOI: Silicon on insulator
SLM: Spatial light modulator
MMA: Methyl methacrylate
DPP: Diphenylphthalate
THF: Tetrahydrofuran
CCD: Charge-coupled device
MMA: Methyl methacrylate
